# A Proposal for a Classification Guiding the Selection of Appropriate Antibiotic Therapy for Intra-Abdominal Infections

**DOI:** 10.3390/antibiotics11101394

**Published:** 2022-10-12

**Authors:** Massimo Sartelli, Francesco Cristini, Federico Coccolini, Francesco Maria Labricciosa, Walter Siquini, Fausto Catena

**Affiliations:** 1 Department of Surgery, Macerata Hospital, 62100 Macerata, Italy; 2 Infectious Disease Department, Rimini Hospital, 47926 Rimini, Italy; 3 General, Emergency and Trauma Department, Pisa University Hospital, 56126 Pisa, Italy; 4 Global Alliance for Infections in Surgery, 62100 Macerata, Italy; 5 General and Emergency Surgery Department, Bufalini Hospital, 47521 Cesena, Italy

**Keywords:** intra-abdominal infections, antimicrobial therapy, antimicrobial prescription

## Abstract

Adequately controlling the source of infection and prescribing appropriately antibiotic therapy are the cornerstones of the management of patients with intra-abdominal infections (IAIs). Correctly classifying patients with IAIs is crucial to assessing the severity of their clinical condition and deciding the strategy of the treatment, including a correct empiric antibiotic therapy. Best practices in prescribing antibiotics may impact patient outcomes and the cost of treatment, as well as the risk of “opportunistic” infections such as *Clostridioides difficile* infection and the development and spread of antimicrobial resistance. This review aims to identify a correct classification of IAIs, guiding clinicians in the selection of the best antibiotic therapy in patients with IAIs.

## 1. Introduction

Intra-abdominal infections (IAIs) are an important source of patient morbidity and may be associated with poor outcomes. Treatment of patients with IAIs has been described in the literature to have satisfactory results if the management is adequate. In many studies, inclusion criteria limit the enrolment of critically ill patients, while in observational studies enrolling patients with no restrictions in inclusion criteria the mortality rate is higher. The Complicated Intra-Abdominal Infection Observational (CIAO) study, the Complicated Intra-Abdominal Infection Observational Worldwide (CIAOW) study, and the World Society of Emergency Surgery (WSES) Complicated Intra-Abdominal Infections Score Study (WISS) study showed mortality rates of 7.5%, 10.5%, and 9.2%, respectively [1,2,3].

Due to restrictive criteria, clinical trials usually over-represent patients who have perforated appendicitis, and patients enrolled in clinical trials have often a high likelihood of survival, because trial eligibility criteria usually restrict the inclusion of patients with serious comorbidities that can increase the death rate of patients [4].

Patients with IAIs should be classified correctly and stratified into low-risk and high-risk groups [5]. Many risk factors have been identified in the literature [6]. These can be related to comorbidities, patient characteristics, and physiological changes associated with the infection; to the adequacy and timing of source control; and/or to the likely presence of multidrug-resistant organisms (MDROs), leading to an ineffective initial empiric antimicrobial therapy.

Correctly classifying patients with IAIs is crucial to assessing the severity of their clinical condition and deciding the strategy of the treatment, including an appropriate empiric antibiotic therapy.

Adequate source control and appropriate antibiotic therapy are the cornerstones in the management of IAIs. There is general consensus that source control is pivotal in the management of IAIs, where both controlling the source of infection and controlling the ongoing contamination are very relevant to improving outcomes. Even if not definitively evaluated by randomised control trials, the increase in death and other adverse outcomes associated with inadequate source control demonstrates that it is of primary importance in treating patients with IAIs [7,8].

In patients with IAIs, antibiotic therapy is also important. It aims to eradicate the residual bacterial infection after source control. In addition, some uncomplicated IAIs (as in the case of some uncomplicated cases of acute appendicitis and acute cholecystitis) can now be treated only with antibiotic therapy. Best practices in prescribing antibiotics may influence patient outcomes and the cost of treatment, as well as the risk of “opportunistic” infections such as *Clostridioides difficile* and the spread of antibiotic resistance in the individual patient and the broader environment. There is evidence that inappropriate empiric antibiotic therapy is associated with treatment failure and poor patient outcomes [9,10,11,12].

The appropriateness of the initial empiric antibiotic therapy prescribed for IAIs is crucial, because at least 24–48 h is required to obtain standard microbiological data to target the antibiotic therapy. Core components of antibiotic prescription practices include the following: adequacy of empiric antibiotic therapy; timing of antibiotic therapy; optimisation of pharmacokinetic/pharmacodynamic (PK/PD) parameters; length of treatment; and reassessment of antibiotic therapy based on the microbiological results and susceptibility testing.

In 2016, the WSES published the executive summary of a consensus conference held on July 23, 2016, in Dublin, Ireland, covering all aspects of the management of IAIs [13]. The first recommendation states that the anatomical extent of infection, the presumed pathogens involved, any individual patient risk factors for difficult-to-treat pathogens, and the patient’s clinical condition should always be assessed independently in classifying patients with IAIs.

This review was designed to identify a correct classification of IAIs, guiding clinicians in the selection of the best antibiotic therapy for patients with IAIs.

## 2. Anatomical Extent of Infection

IAIs represent a heterogeneous group of infections of abdominal origin, ranging from simple acute appendicitis to more complex diffuse peritonitis. A universally accepted classification divides intra-abdominal infections into uncomplicated and complicated cases [14,15].

Uncomplicated IAIs are those infections that, originating from an abdominal organ, remain confined to the same organ, without extending to the peritoneum. In general, patients with uncomplicated IAIs can undergo only surgery or only antibiotic therapy (as in the case of the conservative treatment of uncomplicated acute appendicitis or acute cholecystitis) [16,17,18]. Moreover, in patients with uncomplicated IAIs, when source control is managed adequately, postoperative antibiotics are not necessary [16]. Uncomplicated acute left colonic diverticulitis, in which clinical observation without antibiotic therapy is generally suggested, is excluded from this classification [19].

Complicated IAIs are those infections that, originating from an abdominal organ, extend into the peritoneum, giving rise to peritonitis. Patients with complicated IAIs always require both antibiotic therapy and source control [20]. Nonetheless, there are borderline intra-abdominal conditions that are difficult to categorise as complicated or uncomplicated IAIs, such as localised colonic diverticulitis or periappendiceal phlegmon, which are complicated IAIs but may be managed with antibiotic therapy alone, without a source control procedure.

Patients with complicated IAIs are characterised as manifesting secondary peritonitis. Depending on the peritoneal extent of the infectious process, complicated IAIs are divided into localised and generalised.

Complicated IAIs are localised when the extent of the infectious process is contained by the peritoneal defence mechanisms. They are common in patients with complicated diverticulitis and appendicitis that, although evolving into perforation, can contain the infectious process through an effective peritoneal reaction and with local defence mechanisms, forming a circumscribed secondary acute peritonitis.

Complicated diffuse IAIs, on the other hand, represent the consequence of a massive contamination of the entire peritoneal cavity, following the inability of the patient’s peritoneal defence mechanisms to limit the extent of the intra-abdominal infectious process, forming a secondary acute diffuse peritonitis.

In patients with complicated IAIs, when source control is complete, a short course (3–5 days) of postoperative therapy is generally suggested. This has been confirmed by a prospective control trial (STOP-IT) published in 2015 [21]. The study randomised 260 patients with IAIs undergoing adequate source control and receiving antibiotic therapy until 2 days after the resolution of physiological abnormalities such as fever, leukocytosis, and ileus, with a maximum of 10 days (control group), and 258 receiving a fixed course of antibiotics (experimental group) for 4  ±  1 days. In patients with complicated IAIs undergoing an adequate source control procedure, the outcomes after a fixed duration of antibiotic therapy of approximately 4 days were similar to those after a longer course of antibiotics of approximately 8 days extending until after the resolution of physiological abnormalities. Among the enrolled patients, most of them were not severely ill.

Patients who have ongoing signs of infection or systemic signs of inflammation beyond 5–7 days of antibiotic treatment should warrant a diagnostic investigation to address an ongoing source of infection or failure of antibiotic therapy, and to determine whether a re-laparotomy is necessary.

The limit of this classification is that it does not really describe the complexity of the patient and can create confusion by mixing elements of the extension of the infectious process and severity of the disease expression [22]. On the other hand, in its simplicity, this classification has the advantage of classifying the extension of the infection by identifying which patients always need both antibiotic therapy and source control.

Finally, patients with complicated IAIs may be characterised as manifesting tertiary peritonitis. There is much less agreement regarding what is meant by tertiary peritonitis. It is typical of critically ill or immunocompromised patients and is often associated with multidrug-resistant organisms (MDROs). It is typically associated with poor patient outcomes and is generally considered to be a distinct form of peritonitis [23,24,25]. Tertiary peritonitis is generally described as a persistent or recurrent peritonitis occurring >48 h after apparently adequate surgical source control. It is an evolution of secondary peritonitis and should not be considered as a distinct entity. The term “ongoing peritonitis” [26] or “persistent peritonitis” [27] may better indicate the physiopathology of this clinical condition.

## 3. Presumed Pathogens Involved and Individual Patient Risk Factors for Difficult-to-Treat Pathogens

A classification according to the place where the patient contracts the infection divides the IAIs into community-acquired (CA-IAIs), if acquired in the community, or hospital-acquired (HA-IAIs), if acquired in a hospital or in health residences.

“Healthcare-associated infection” (HCAIs) is a commonly used term for describing infections acquired during the course of receiving healthcare, including not only hospital-acquired infections but also infections in patients living in long-term care facilities, recently hospitalised, or undergoing recent aggressive medical therapies. However, in the setting of HCAIs, there are few data regarding the concept of HCAIs as opposed to hospital-acquired infections [28].

Differentiating patients with CA-IAIs from patients with HA-IAIs allows the identification of patients with increased likelihood of IAIs caused by MDROs.

Unlike CA-IAIs, HA-IAIs show a poorer prognosis and require more aggressive antibiotic therapy. HA-IAIs usually occur after a period of hospitalisation—often after surgery, and in patients already treated with antibiotic therapy. From a microbiological point of view, CA-IAIs are characterised by the presence of bacteria usually residing in the gastrointestinal tract, and are therefore generally predictable [1,2]. HA-IAIs often involve multidrug-resistant and unpredictable bacteria, in terms of both the bacterial species involved and their sensitivity to antibiotics.

Among patients with HA-IAIs, those with postoperative peritonitis may be associated with increased mortality due to underlying patient comorbidity, atypical presentation due to non-specific clinical signs, and risk factors for acquiring MDROs and *Candida* spp. infections [29,30,31].

Antibiotics for empiric treatment of CA-IAIs should cover enteric Gram-negative aerobic and facultative bacteria, enteric Gram-positive streptococci, and obligate anaerobic bacilli such as *Bacteroides fragilis* (especially for IAIs derived from the distal small bowel, appendix, or colon) [32]. The most commonly isolated Gram-negative facultative organism is *Escherichia coli* [1,2].

Due to the increasing prevalence of *Enterobacterales* resistant to amoxicillin/clavulanate observed in community-acquired infections, caution should be taken in using amoxicillin/clavulanate in settings with a high local rate of resistance to this antibiotic.

However, most *Enterobacterales* remain susceptible to piperacillin/tazobactam. Its broad-spectrum activity makes it an attractive option in the management of CA-IAIs, especially in critically ill patients or in patients with CA-IAIs with other risk factors of adverse outcomes—including advanced age (70 years of age or older); presence of malignant disease; major compromise of cardiovascular, hepatic, or renal function; and hypoalbuminemia [6].

Most isolates of *E. coli* and other *Enterobacterales* in CA-IAIs remain susceptible to third-generation cephalosporins. Among third-generation cephalosporins, cefotaxime or ceftriaxone in association with metronidazole may be options for empiric therapy of CA-IAIs in patients with no risk factors for ESBLs. Cefepime is a fourth-generation cephalosporin, with broader-spectrum activity than ceftriaxone. It is poorly hydrolysed by AmpC beta-lactamase, allowing it to be effective against AmpC-producing organisms [15]. For empiric therapy, cefepime must be combined with metronidazole, because it is inactive against anaerobes.

In recent years, fluoroquinolones have been widely used in the treatment of IAIs due to their activity against aerobic Gram-negative bacteria and tissue penetration. The worldwide increase in resistance among *E. coli* and other *Enterobacterales* has limited the use of fluoroquinolones for empiric treatment of CA-IAIs, and they are generally suggested in association with metronidazole and only in non-critically ill patients with allergy to beta-lactam agents.

In the context of complicated IAIs, the main resistance burden is posed by ESBL-producing *Enterobacterales*, which are prevalent in hospital-acquired infections but are also observed in community-acquired infections [33,34]. ESBLs are enzymes able to hydrolyse and inactivate a wide variety of beta-lactams, such as third-generation cephalosporins, penicillins, and aztreonam [35,36]. Most ESBLs of clinical interest are encoded by genes located on plasmids. They are able to carry genes encoding resistance to other classes of antibiotics, including aminoglycosides and fluoroquinolones [36]. Although routine testing for ESBLs is not performed by most microbiology laboratories [37], non-susceptibility to ceftriaxone (MICs ≥ 2 mcg/mL) may be used to confirm ESBL infections. However, recognising ESBLs on the basis of susceptibility to ceftriaxone may present limitations, as bacteria that are not susceptible to ceftriaxone due to mechanisms other than ESBL production may be incorrectly presumed to be ESBL-producers [38,39].

ESBLs generally should not be covered in patients with CA-IAIs with no signs of severity or without risk of treatment failure, except in regions with particular local epidemiological conditions, where there is a high likelihood that ESBL-producing *Enterobacterales* may be components of the infection.

Risk factors of ESBL-producing *Enterobacterales* in CA-IAIs include recent exposure to antibiotics (particularly third-generation cephalosporins or fluoroquinolones) and known colonisation with ESBL producing *Enterobacterales*. These aspects should always be considered in stratifying patients with CA-IAIs to prescribe an adequate empiric therapy.

Carbapenems have been considered the empiric antibiotics of choice for treating patients with ESBL-producing *Enterobacterales*. Group 1 carbapenems include ertapenem—a once-a-day carbapenem sharing the same activity of Group 2 carbapenems against ESBL-producing *Enterobacterales*. However, it is not active against *Pseudomonas aeruginosa* and enterococci [40]. Group 2 includes imipenem/cilastatin, meropenem, and doripenem. Compared to ertapenem, they have activity against non-fermentative Gram-negative bacilli. Unlike meropenem and doripenem, imipenem/cilastatin is active against enterococci that are susceptible to ampicillin.

However, in order to avoid excessive carbapenem use, carbapenem-sparing strategies using other antibiotics—such as piperacillin/tazobactam, an aminoglycoside agent, tigecycline, or eravacycline—should be considered.

The significance of piperacillin/tazobactam for treating ESBL-producing *Enterobacterales* has been a debated issue. Gram-negative bacteria have the ability to concomitantly produce multiple ESBLs as well as AmpC beta-lactamases and can possess other mechanisms of resistance limiting the activity of piperacillin/tazobactam [41]. On the other hand, the activity of piperacillin/tazobactam is influenced by the “inoculum effect”—a laboratory phenomenon described as a significant increase in the MIC of an antibiotic when a great number of bacteria are inoculated [41].

An RCT conducted in patients with ESBL-producing *Enterobacterales* bloodstream infections [42] demonstrated inferior results of piperacillin/tazobactam compared to carbapenems. Although piperacillin/tazobactam is not considered the first-choice antibiotic to treat ESBL-producing *Enterobacterales* [43], it may still be considered a valuable carbapenem-sparing agent in the management of ESBLs in IAIs treated with adequate source control when dealing with fully susceptible bacteria (MIC ≤ 8 mg/L). A high dose (18 g) should be prescribed to optimise PK/PD targeting in critically ill patients [44].

Aminoglycosides have in vitro activity against aerobic Gram-negative bacteria, including ESBL-producing *Enterobacterales*, and act synergistically against certain Gram-positive bacteria.

Because of their serious toxic side effects, including nephrotoxicity and ototoxicity, some authors do not recommend aminoglycosides for the routine empiric treatment of IAIs [6]. They may be reserved for patients with allergies to beta-lactam agents or used in combination with beta-lactam agents [45]. In any case, this class of antibiotics remains an important option to treat Gram-negative bacteria and widen the spectrum of antibiotic therapy when resistant organisms are suspected.

Tigecycline remains a useful option for treating patients with complicated IAIs, due to its favourable activity against anaerobic organisms, enterococci, and ESBLs [46]. It has no in vitro activity against *P. aeruginosa* or certain *Enterobacterales*, including *Proteus* spp. and *Serratia* spp. Excess mortality was observed in patients treated with tigecycline when compared with other antibiotics [47]. Study-level and patient-level analyses demonstrated that, in particular, patients with ventilator-associated pneumonia and baseline bacteraemia were at a higher risk of mortality. A mortality analysis was used to investigate the association of baseline factors with clinical failure and mortality in complicated IAIs and did not suggest that tigecycline was a factor either for failure or for death in phase 3 and 4 comparative clinical trials of tigecycline [48]. Tigecycline should not be considered the first-line option for treating hospital-acquired pneumonia and bacteraemia.

Eravacycline is an antibiotic that is structurally similar to tigecycline. Eravacycline demonstrates broad-spectrum activity against Gram-positive, Gram-negative—including ESBLs-producing *Enterobacterales*—and anaerobic bacteria. Like tigecycline, it is inactive against *P. aeruginosa* [49]. Eravacycline is well-tolerated with nausea and vomiting and, interestingly, is also available for oral administration.

Finally, ceftolozane/tazobactam and ceftazidime/avibactam have shown efficacy in treating patients with IAIs caused by ESBL-producing *Enterobacterales* [50,51]. They may be especially useful in critically ill patients when dealing with isolates exhibiting high MIC values [44]. In settings with a high prevalence of carbapenem-resistant *Enterobacterales* (CRE), ceftazidime/avibactam should be reserved for the treatment of CRE.

Among Gram-negative bacteria, carbapenem-resistant *Enterobacterales* represent a heterogeneous group of bacteria with more potential mechanisms of antibiotic resistance. They are generally divided into carbapenemase-producing *Enterobacterales* and non-carbapenemase-producing *Enterobacterales*. Carbapenemases hydrolyse penicillins, all cephalosporins, beta-lactamase inhibitors, and even carbapenems. The most widespread carbapenemases are *Klebsiella pneumoniae* carbapenemases [52,53,54,55]. These are rapidly emerging as an important source of hospital-acquired infections in many regions of the world. Metallo-beta-lactamases (MBLs) differ from the other beta-lactamases in their requirement of zinc for activity. MBLs are all capable of hydrolysing most beta-lactam agents, including carbapenems, except for the monobactam aztreonam.

Alarming rates of resistance to many antibiotics in health facilities worldwide have been reported for non-fermenting Gram-negative bacteria, including *P. aeruginosa*, *Stenotrophomonas maltophilia*, and *Acinetobacter baumannii.* Non-fermenting Gram-negative bacteria are intrinsically resistant to many antibiotics; moreover, they can acquire additional resistance to other important antibiotic agents. These mechanisms may be present simultaneously and can confer resistance to different classes of antibiotics.

Various mechanisms of resistance have been identified in *P. aeruginosa*, including membrane permeability defects, expression of efflux pumps, and production of antibiotic-hydrolysing enzymes such as AmpC beta-lactamases or carbapenemases. The most prevalent carbapenemases in *P. aeruginosa* are MBLs, such as Verona integron-encoded metallo-β-lactamase (VIM) and imipenemase (IMP) types.

In recent years, several new antibiotics with predominant activity against Gram-negative bacteria have been approved by the U.S. Food and Drug Administration (FDA) and the European Medicines Agency (EMA). Meropenem/vaborbactam, ceftazidime/avibactam, and imipenem/cilastatin/relebactam are the options for carbapenemase-producing *K. pneumoniae* infections [56,57,58,59,60,61,62,63,64,65].

The lack of in vitro activity of ceftazidime/avibactam against metallo-beta-lactamase (MBL) and the observation that many MBL-producing infections can coproduce other beta-lactamases—including ESBLs, AmpC, and OXA-48—suggest a potential effect of combining ceftazidime/avibactam with aztreonam, which is not hydrolysed by MBLs [66,67,68].

Ceftazidime/avibactam is the preferred treatment option for OXA-48-like-producing *Enterobacterales*. It has no activity against anaerobic bacteria.

Ceftazidime/avibactam in combination with aztreonam or cefiderocol [66] as monotherapy are the preferred treatment option for MBL-producing *Enterobacterales*.

Due to its epithelial lining fluid penetration, meropenem-vaborbactam should be used as first therapeutic choice in patients with ventilator-associated pneumonia due to KPC-producing *Enterobacterales*.

Cefiderocol appears to be effective in vitro against all resistance phenotypes of *P. aeruginosa*, including MBLs. Cefiderocol is also effective in vitro against extreme drug resistant *A. baumannii*. Despite these promising early data, a recent clinical trial did not support the higher effectiveness of cefiderocol in the patient subset with *A. baumannii* infections [69]. Therefore, further clinical data are needed to better understand the role of this novel option [70].

Local epidemiological data for selecting an adequate empiric antibiotic therapy in patients with HA-IAIs at risk of infection with drug-resistant Gram-negative bacteria can be useful to define the appropriate antibiotic approach.

Among Gram-positive bacteria, the pathogenicity of enterococci in IAIs has been a debated issue [71,72,73,74], also hypothesising a synergistic effect with other bacteria such as *E. coli* and anaerobes [75,76,77]. Some studies have demonstrated poor outcomes in patients with HA-IAIs, especially in critically ill patients [72,73]. In this setting, coverage against enterococci should be always considered. Although *Enterococcus* isolation was not found to be an independent risk factor for the composite outcomes in non-critically ill patients in a post hoc analysis of the STOP-IT trial database [74], patients who had previously received cephalosporins or other antibiotics for enterococci, immunocompromised patients, and patients with valvular heart disease or prosthetic intravascular materials were also considered to need coverage against enterococci [32].

Most strains of *Enterococcus faecalis* are susceptible to ampicillin. In patients with IAIs, *Enterococcus faecium* is increasingly encountered—particularly in patients with HA-IAIs. In contrast to *E. faecalis*, nearly all strains of *E. faecium* are resistant to ampicillin [78,79]. First-line treatment of vancomycin-susceptible strains of *E. faecium* includes vancomycin or other glycopeptides, such as teicoplanin. The incidence of vancomycin-resistant *E. faecium* (VRE) is increasing. The acquisition of glycopeptide resistance by enterococci can seriously affect the treatment and control of these organisms in patients with hospital-acquired infections [71]. Linezolid and daptomycin may be used for the management of suspected or proven infections caused by VRE.

*Staphylococcus aureus* is not common in patients with IAIs. It is rarely isolated in patients with CA-IAIs, but it may be found to some extent in patients with HA-IAIs and may be resistant to methicillin (MRSA). Patients with HA-IAIs, those colonised with MRSA, and those with multiple healthcare-associated risk factors for MRSA colonisation—including previous hospitalisation or surgery—and significant recent exposure to antibiotics may be considered at risk of infection by MRSA. Glycopeptides such as vancomycin may be used for the treatment of IAIs to provide therapy for suspected or proven infections caused by vancomycin-susceptible MRSA. *S. aureus* strains with increased resistance to vancomycin—known as vancomycin-intermediate-resistant *S. aureus* (VISA), exhibiting an MIC of 4–8 µg/mL, and *S. aureus* completely resistant to vancomycin (VRSA), exhibiting an MIC ≥ 16 µg/mL—are increasing [80]. Linezolid and daptomycin may be used for the management of suspected or proven infections caused by VRSA.

## 4. Patients’ Clinical Condition

Grading of the clinical severity of patients with IAIs has been well described by the sepsis definitions [81]. A prospective observational study involving 180 consecutive patients with secondary generalised peritonitis (community-acquired and postoperative) showed that patients with septic shock had a higher mortality rate than patients without shock (35% versus 8%).

The data from the WISS study [3] showed that mortality was significantly affected by sepsis and that mortality rates increased in patients developing organ dysfunction and septic shock. Sepsis-related mortality was as follows: no sepsis = 1.2%, sepsis only = 4.4%, severe sepsis = 27.8%, and septic shock = 67.8%.

In February 2016, a proposal for new definitions for sepsis (Sepsis-3) was published in the *Journal of the American Medical Association* (JAMA) [82]. These definitions updated the previous sepsis definitions [83,84]. The new definitions were intended to provide a standardised classification facilitating clinical care, reporting, and research. The Sepsis-3 definitions classify sepsis as life-threatening organ dysfunction caused by a dysregulated response of the host to infection, emphasising the potential lethality and the need for urgent recognition. Organ dysfunction is determined by an increase in the Sequential Organ Failure Assessment (SOFA) score of 2 points or more that is associated with a risk of in-hospital mortality greater than 10%. Septic shock is defined as a subset of sepsis and is clinically characterised by the requirement of vasopressor agents to maintain a mean arterial pressure of 65 mmHg or more and a serum lactate level above 2 mmol/L (>18 mg/dL) in the absence of hypovolemia.

Human immune responses to an infectious focus can vary greatly from individual to individual. Some individuals greatly overreact to infection, producing a massive cytokine storm leading to multi-organ failure. On the other hand, some individuals have little response to the same infection. Identifying patients overreacting to the infection as soon as possible and correcting the underlying dysfunction may improve patient outcomes [85,86]. An insufficient or otherwise inadequate antibiotic regimen is one of the variables more strongly associated with unfavourable outcomes in critically ill patients [87].

These findings have been confirmed in a recent meta-analysis demonstrating reduced mortality and significantly shorter hospital lengths of stay, with corresponding reductions in hospital costs, in patients receiving early appropriate versus inappropriate antibiotic therapy for severe bacterial infection [88].

The reduction in mortality associated with early administration of antibiotics is strongest in patients with septic shock. Studies have reported a strong association between time to antibiotics and mortality in patients with septic shock, but a weaker association in patients with sepsis but without septic shock.

A recent review suggests that a reasonable timeframe would be no later than 3–5 h after the onset of infection, but immediately for patients with septic shock [89]. Based on the best available data, there is a strong relationship between timing of antibiotic administration and mortality in patients with septic shock, but a less pronounced relationship in patients with sepsis and without shock [90].

The 2021 Surviving Sepsis Campaign guidelines recommend the immediate administration of antibiotics in patients with possible septic shock or a high likelihood of sepsis—ideally within 1 h from the time when sepsis was first recognised. In patients with possible sepsis without shock, the guidelines suggest a time-limited course of rapid investigation and administering antibiotics within 3 h of recognition [91].

Considering the spread of MDROs in many regions of the world and the association between delaying appropriate antibiotic therapy and worse outcomes in patients with sepsis and septic shock, the initial administration of a broad-spectrum regimen including at least one effective agent active against the suspected or proven offending organism is crucial.

Antibiotic de-escalation is now considered a key point of antimicrobial stewardship programs in intensive care units (ICUs) [92]. However, its real efficacy has been debated and has not been convincingly demonstrated in a clinical setting [93]. Even if it appears beneficial, it may have some side effects, including its use as a justification of unrestricted broad-spectrum empiric antibiotic therapy and the exposure of patients to multiple, sequential antibiotics with unwanted effects on the microbiome.

Montravers et al. [94] evaluated the characteristics and outcomes of antimicrobial de-escalation in patients with HA-IAIs, demonstrating that de-escalation is a feasible option in these patients, even if multidrug-resistant non-fermenting Gram-negative organisms may limit its implementation in the setting of IAIs. When treating patients with sepsis of abdominal origin, clinicians should be aware that antibiotic pharmacokinetics may be modified by the pathophysiology of sepsis, and should always consider the pathophysiological and immunological status of the patient [94]. In fact, antibiotics are subject to changes in PK/PD parameters in patients with sepsis and septic shock, where low antibiotic levels may be too low, risking clinical failure, or too high, risking toxicity.

The “dilution effect” should be considered for hydrophilic antibiotics—including beta-lactam agents, aminoglycosides, and glycopeptides—that selectively distribute to the extracellular space [95]. Low plasma antibiotic concentrations can contribute to lower levels in peritoneal fluid, with potentially reduced antibiotic activity in the target tissues.

Higher than standard loading doses of beta-lactam agents, aminoglycosides, fosfomycin, or glycopeptides should be administered to ensure optimal antibiotic concentration at the infection site in patients with sepsis or septic shock.

Once appropriate initial loading is administered, it is crucial to reassess the antibiotic regimen daily, because pathophysiological changes may occur, significantly affecting antibiotic distribution in critically ill patients. Lower dosages of renally excreted antibiotics should be administered in patients with impaired renal function, while higher dosages of renally excreted antibiotics are needed for optimal exposure in patients with glomerular hyperfiltration. In critically ill patients, plasma creatinine is an unreliable marker of renal function [95].

Adequate dosing is associated with the concept of time-dependent versus concentration-dependent killing. Beta-lactam agents have time-dependent activity, exerting optimal activity when antibiotic concentrations are maintained above the minimum inhibitory concentration (MIC) [95]. For beta-lactam agents, prolonged or continuous infusions have been advocated in order to maximise the time for which the drug concentration exceeds the MIC. However, a randomised controlled trial comparing continuous versus intermittent infusion of piperacillin/tazobactam in the specific setting of complicated IAIs [96] did not show different outcomes.

These results should not be generalisable to critically ill patients and patients with infections caused by less susceptible bacteria with high MIC values, where prolonged infusion with a beta-lactam agent seems to have more advantages than in patients with infections caused by sensitive bacteria [97,98].

Although there is insufficient evidence to reach definitive strong conclusions, two meta-analyses have demonstrated reduced short-term mortality with prolonged infusion of beta-lactam agents in critically ill patients [99,100]. No trials have assessed the undesirable effects of continuous infusion. However, meta-analyses evaluating the effects of prolonged infusion in subgroups of stratified patients are needed to identify the subgroup of patients benefiting most from prolonged infusion [101].

Interestingly, a recent meta-analysis evaluating prolonged versus intermittent beta-lactam antibiotics in patients with sepsis or septic shock [102] showed favourable effects of prolonged infusion in studies published in 2015 or after.

On the other hand, antibiotics such as aminoglycosides have concentration-dependent activity and should be administered in a once-daily manner (or with the least possible number of daily administrations) in order to achieve high peak plasma concentrations [95]. Nephrotoxicity related to aminoglycosides is caused by direct damage to the renal cortex and its uptake saturation. Thus, a correct dosing strategy may reduce the renal cortex’s exposure to aminoglycosides, reducing the risk of nephrotoxicity [103].

Another group of patients at high risk of treatment failure are immunocompromised patients. Immunosuppressive status includes congenital conditions such as T- or B-cell defects and macrophage dysfunctions, as well as acquired conditions such as infections with human immunodeficiency virus (HIV) developing acquired immunodeficiency syndrome, haematological malignancies or solid malignancies needing chemotherapy, and solid organ transplantation patients or those with inflammatory disease/rheumatologic disease needing immunomodulatory agents [104]. The management of these patients with IAIs should be individualised and needs an appropriate stratification to plan the most appropriate therapeutic pathways [105].

## 5. Conclusions

The anatomical extent of the infection, the presumed pathogens involved, the risk factors for major resistance patterns, and the patient’s clinical condition should be assessed independently so as to classify patients. This allows the stratification of patients with IAIs, guiding physicians in providing the appropriate antibiotic strategy.

To assess the anatomical extent of the infection, physicians should always determine whether the IAI is complicated or uncomplicated. Unlike patients with complicated IAIs, who always require both antibiotic therapy and source control, patients with uncomplicated IAIs do not require postoperative antibiotics when source control is managed adequately.

To assess the presumed pathogens involved and any risk factors for major resistance patterns, physicians should always determine whether the IAI is community-acquired or hospital-acquired. HA-IAIs often involve multidrug-resistant and unpredictable bacteria, in terms of both the bacterial species involved and their sensitivity to antibiotics. Among patients with HA-IAIs, those with postoperative peritonitis may be associated with increased mortality due to underlying patient comorbidity, atypical presentation due to non-specific clinical signs, and risk factors for acquiring MDROs and *Candida* spp. infections.

In order to assess the grading of the clinical severity of patients, physicians should always determine the patients’ sepsis condition. Identifying patients overreacting to the infection as soon as possible and administering antibiotics may improve patient outcomes, especially in patients with septic shock. The 2021 Surviving Sepsis Campaign guidelines recommend the immediate administration of antibiotics in patients with possible septic shock or a high likelihood of sepsis—ideally within 1 h from the time when sepsis was first recognised. In patients with possible sepsis without shock, the guidelines suggest a time-limited course of rapid investigation and administering antibiotics within 3 h of recognition [91].

In Table 1, Table 2, Table 3, Table 4, Table 5, Table 6, Table 7 and Table 8, proposals for antibiotic regimens for the treatment of IAIs are illustrated.

Abdominal tuberculosis (TB) is an increasingly common disease posing diagnostic challenges because of the non-specific features of the disease. Abdominal TB is a type of extrapulmonary tuberculosis that involves the abdominal organs, such as the intestines, peritoneum, and abdominal lymph nodes. It can occur either in isolation or along with a primary focus (such as the lungs) in patients with disseminated tuberculosis. This review does not refer to abdominal TB, which will be considered individually in an upcoming review.

## Figures and Tables

**Table 1:** Patients with community-acquired intra-abdominal infection without sepsis or septic shock.

Empiric Antibiotic Regimens; Normal Renal Function
One of the following intravenous antibiotics: Amoxicillin/clavulanate 2.2 g q8h ^1^ Ceftriaxone 2 g every q24h + metronidazole 500 mg q8h Cefotaxime 2 g every 8 h + metronidazole 500 mg q8h Piperacillin/tazobactam 4 g/0.5 g q6h ^2^
In patients with beta-lactam allergy, a fluoroquinolone-based regimen: Ciprofloxacin 400 mg every q8/12h + metronidazole 500 mg q8h
In patients with beta-lactam allergy, an aminoglycoside regimen: Amikacin 15–20 mg/kg q24h + metronidazole 500 q8h
In patients at high risk of infection with community-acquired ESBL-producing *Enterobacterales*, one of the following antibiotics: Tigecycline 100 mg LD, then 50 mg every q12h (carbapenem-sparing strategy) Ertapenem 1 g q24h

^1^ Its use should be avoided if *Enterobacterales* local rate of resistance >20%. ^2^ In patients with advanced age (70 years of age or greater); presence of malignant disease; major compromise of cardiovascular, hepatic, or renal function; and/or hypoalbuminemia.

**Table 2:** Patients with community-acquired intra-abdominal infection with sepsis or septic shock.

Empiric Antibiotic Regimens; Normal Renal Function
One of the following intravenous antibiotics: Piperacillin/tazobactam 6 g/0.75 g LD then 4 g/0.5 g q6h or 16 g/2 g by continuous infusion.
In patients with documented beta-lactam allergy, an aminoglycoside regimen: Amikacin 15–20 mg/kg q24h + metronidazole 500 mg q8h
In patients at high risk of infection with community-acquired ESBL-producing *Enterobacterales*, one of the following antibiotics: Meropenem 1 g q8h (only in patients with septic shock) ^1^ Doripenem 500 mg q8h (only in patients with septic shock) ^1^ Imipenem/cilastatin 500 mg q6h (only in patients with septic shock)

^1^ Meropenem and doripenem have no in vitro activity against enterococci that are susceptible to ampicillin.

**Table 3:** Patients with hospital-acquired IAIs without sepsis or septic shock.

Empiric Antibiotic Regimens; Normal Renal Function
One of the following intravenous antibiotics: Tigecycline 100 mg LD, then 50 mg every 12 h (not active against *P. aeruginosa*) Eravacycline 1 mg/kg q12 h (not active against *P. aeruginosa*) + Piperacillin/tazobactam 4.5 q6h
In patients with documented beta-lactam allergy: Amikacin 15–20 mg/kg q24h
In patients with high risk for invasive candidiasis: + Fluconazole 800 mg LD then 400 mg every 24 h

**Table 4:** Patients with hospital-acquired intra-abdominal infection with sepsis or septic shock.

Empiric Antibiotic Regimens; Normal Renal Function
One of the following intravenous antibiotics: Meropenem 1 g q8h Doripenem 500 mg q8h Imipenem/cilastatin 500 mg q6h + One of the following intravenous antibiotics: Vancomycin 25–30 mg/kg LD then 15–20 mg/kg/q8 h Teicoplanin 12 mg/kg every 12 h 3 LDs then 12 mg/kg q24 h
In patients with high risk for invasive candidiasis, add one of the following antifungal agents: Caspofungin 70 mg LD, then 50 mg q24h Anidulafungin 200 mg LD, then 100 q24h Micafungin 100 mg q24h Amphotericin B liposomal 3 mg/kg q24h
In patients with suspected or proven infection with difficult-to-treat ^1^ non-metallo-beta-lactamase-producing *P. aeruginosa*, consider the use of antibiotic combinations with: Ceftolozane/tazobactam (1.5 g q8h), ceftazidime-avibactam (2.5 g q8h), and imipenem/cilastatin-relebactam (1.25 g q6h)
In patients with suspected or proven infection with carbapenemase-producing *K. pneumoniae* and MDR (non-metallo-beta-lactamase-producing) *P. aeruginosa*, consider the use of antibiotic combinations with: Ceftazidime-avibactam (2.5 g q8h), meropenem-vaborbactam (4 g 8qh), and imipenem/cilastatin-relebactam (1.25 g q6h)
In patients with suspected or proven infection with metallo-beta-lactamase-producing bacteria (i.e., NDM, VIM, IMP), consider the use of antibiotic combinations with: Ceftazidime-avibactam (2.5 g q8h) + aztreonam (2 g q8h) or cefiderocol (2 g q8h)
In patients with suspected or proven infection with vancomycin-resistant enterococci (VRE)—including patients with previous enterococcal infection or colonisation, immunocompromised patients, patients with long ICU stay, or patients with recent vancomycin exposure—consider the use of antibiotic combinations with: Linezolid (600 q 12h) or daptomycin (10–12 mg/kg q24h) ^2^

^1^ Difficult-to-treat is defined as *P. aeruginosa* that exhibits no susceptibility to any of the following: piperacillin/tazobactam, ceftazidime, cefepime, aztreonam, meropenem, imipenem/cilastatin, ciprofloxacin, and levofloxacin. ^2^ Approved at the dosage of 4 mg/kg/24 h, it is currently used at higher dosages.

**Table 5:** Patients with IAIs with known colonisation (infection) by KPC-producing *Enterobacterales*.

Oriented Antibiotic Regimens; Normal Renal Function
One of the following intravenous antibiotics ^1^: Ceftazidime/avibactam 2.5 g q8h + tigecycline 100 mg LD, then 50 mg every 12 h Ceftazidime/avibactam 2.5 g q8h + metronidazole 500 mg q8h Meropenem/vaborbactam 4 g 8qh infused in three hours Imipenem/cilastatin/relebactam 1.25 g q6h + One of the following intravenous antibiotics (not for combinations with tigecycline): Vancomycin 25–30 mg/kg LD then 15–20 mg/kg/q8 h Teicoplanin 12 mg/kg every 12 h 3 LDs then 12 mg/kg q24 h
In patients at high risk of invasive candidiasis, add one of the following antifungal agents: Caspofungin 70 mg LD, then 50 mg q24h Anidulafungin 200 mg LD, then 100 q24h Micafungin 100 mg q24h Amphotericin B liposomal 3 mg/kg q24h

KPC: *Klebsiella pneumonia* carbapenemase. ^1^ The microorganism is known to be sensitive to the chosen beta-lactam.

**Table 6:** Patients with IAIs with known colonisation (infection) by MBL-producing *Enterobacterales*.

Oriented Antibiotic Regimens; Normal Renal Function
One of the following intravenous antibiotics: Ceftazidime/avibactam 2.5 g q8h + aztreonam 2 g q8h + tigecycline 100 mg LD, then 50 mg every 12 h Ceftazidime/avibactam 2.5 g q8h + aztreonam 2 g q8h + metronidazole 500 mg q8h Cefiderocol 2 g q8h + tigecycline 100 mg LD, then 50 mg every 12 h Cefiderocol 2 g q8h + metronidazole 500 mg q8h
In patients at high risk of invasive candidiasis, add one of the following antifungal agents: Caspofungin 70 mg LD, then 50 mg q24h Anidulafungin 200 mg LD, then 100 q24h Micafungin 100 mg q24h Amphotericin B Liposomal 3 mg/kg q24h

MBL: metallo-beta-lactamase.

**Table 7:** Patients with IAIs with known colonisation by MBL-producing *P. aeruginosa*.

Oriented Antibiotic Regimens; Normal Renal Function
One of the following intravenous antibiotics: Cefiderocol 2 g q8h + tigecycline 100 mg LD, then 50 mg every 12 h Cefiderocol 2 g q8h + metronidazole 500 mg q8h Meropenem 2 g q8h + fosfomycin 4 g q6h + One of the following intravenous antibiotics (not for combinations with tigecycline or fosfomycin): Vancomycin 25–30 mg/kg LD then 15–20 mg/kg/q8 h Teicoplanin 12 mg/kg every 12 h 3 LDs then 12 mg/kg q24 h
In patients at high risk of invasive candidiasis, add one of the following antifungal agents: Caspofungin 70 mg LD, then 50 mg q24h Anidulafungin 200 mg LD, then 100 q24h Micafungin 100 mg q24h Amphotericin B liposomal 3 mg/kg q24h

MBL: metallo-beta-lactamase.

**Table 8:** Patients with IAIs with known colonisation by carbapenem-resistant *A. baumannii*.

Oriented Antibiotic Regimens; Normal Renal Function
One of the following intravenous antibiotics: Cefiderocol 2 g q8h + tigecycline 100 mg LD, then 50 mg q12h Cefiderocol 2 g q8h + metronidazole 500 mg q8h Fosfomycin 4 g q6h + ampicillin/sulbactam 6/3 g q8h + One of the following intravenous antibiotics (not for combinations with tigecycline or fosfomycin): Vancomycin 25–30 mg/kg LD then 15–20 mg/kg/q8h Teicoplanin 12 mg/kg every 12 h 3 LDs then 12 mg/kg q24h
In patients at high risk of invasive candidiasis, add one of the following antifungal agents: Caspofungin 70 mg LD, then 50 mg q24h Anidulafungin 200 mg LD, then 100 q24h Micafungin 100 mg q24h Amphotericin B liposomal 3 mg/kg q24h
